# Optimized strategy for real-time qPCR detection of *Onchocerca volvulus* DNA in pooled *Simulium sp*. blackfly vectors

**DOI:** 10.1371/journal.pntd.0011815

**Published:** 2023-12-14

**Authors:** Mary Doherty, Jessica R. Grant, Nils Pilotte, Sasisekhar Bennuru, Kerstin Fischer, Peter U. Fischer, Sara Lustigman, Thomas B. Nutman, Kenneth Pfarr, Achim Hoerauf, Thomas R. Unnasch, Hassan K. Hassan, Samuel Wanji, Patrick J. Lammie, Eric Ottesen, Charles Mackenzie, Steven A. Williams

**Affiliations:** 1 Department of Biological Sciences, Smith College, Northampton, Massachusetts, United States of America; 2 Department of Biological Sciences, Quinnipiac University, Hamden, Connecticut, United States of America; 3 Laboratory of Parasitic Diseases, National Institute of Allergy and Infectious Diseases, Bethesda, Maryland, United States of America; 4 Department of Medicine, Washington University School of Medicine, St. Louis, Missouri, United States of America; 5 Laboratory of Molecular Parasitology, Lindsley F. Kimball Research Institute, New York Blood Center, New York, New York, United States of America; 6 Institute of Medical Microbiology, Immunology and Parasitology, University Hospital Bonn, Bonn, Germany; 7 German Center for Infection Research (DZIF), Partner-Site Bonn-Cologne, Bonn, Germany; 8 Center for Global Health Infectious Disease Research, University of South Florida, Tampa, Florida, United States of America; 9 Parasite and Vectors Research Unit, Department of Microbiology and Parasitology, University of Buea, Buea, Cameroon; 10 Research Foundation in Tropical Diseases and the Environment, Buea, Cameroon; 11 NTD-SC, Task Force for Global Health, Atlanta, Georgia, United States of America; 12 RLMF, The END Fund, New York, New York, United States of America; 13 Molecular and Cellular Biology Program, University of Massachusetts, Amherst, Massachusetts, United States of America; University of Liverpool, UNITED KINGDOM

## Abstract

**Background:**

*Onchocerca volvulus* is a filarial parasite that is a major cause of dermatitis and blindness in endemic regions primarily in sub-Saharan Africa. Widespread efforts to control the disease caused by *O*. *volvulus* infection (onchocerciasis) began in 1974 and in recent years, following successful elimination of transmission in much of the Americas, the focus of efforts in Africa has moved from control to the more challenging goal of elimination of transmission in all endemic countries. Mass drug administration (MDA) with ivermectin has reached more than 150 million people and elimination of transmission has been confirmed in four South American countries, with at least two African countries having now stopped MDA as they approach verification of elimination. It is essential that accurate data for active transmission are used to assist in making the critical decision to stop MDA, since missing low levels of transmission and infection can lead to continued spread or recrudescence of the disease.

**Methodology/Principal findings:**

Current World Health Organization guidelines for MDA stopping decisions and post-treatment surveillance include screening pools of the *Simulium* blackfly vector for the presence of *O*. *volvulus* larvae using a PCR-ELISA-based molecular technique. In this study, we address the potential of an updated, practical, standardized molecular diagnostic tool with increased sensitivity and species-specificity by comparing several candidate qPCR assays. When paired with heat-stable reagents, a qPCR assay with a mitochondrial DNA target (OvND5) was found to be more sensitive and species-specific than an O150 qPCR, which targets a non-protein coding repetitive DNA sequence. The OvND5 assay detected 19/20 pools of 100 blackfly heads spiked with a single L3, compared to 16/20 for the O150 qPCR assay.

**Conclusions/Significance:**

Given the improved sensitivity, species-specificity and resistance to PCR inhibitors, we identified OvND5 as the optimal target for field sample detection. All reagents for this assay can be shipped at room temperature with no loss of activity. The qPCR protocol we propose is also simpler, faster, and more cost-effective than the current end-point molecular assays.

## Author summary

*Onchocerca volvulus* is a nematode parasite that is the causative agent of river blindness in Africa, the Americas and Yemen and is transmitted by blackflies of the genus *Simulium*. Efforts to reduce and eliminate this neglected tropical disease focus on mass drug administration (MDA) with ivermectin and require reliable and accurate diagnostic assays to monitor the progress of once or twice-yearly treatments. One important measure of the impact of MDA is to screen pools of vector flies collected in endemic areas for the presence of L3-stage (infective) larvae. Since dissection of individual flies and microscopic examination to search for larvae in the flies is very time-consuming and results in many false negative and false positive results (due to morphologically similar larvae of closely related animal parasites), DNA-based PCR assays have proven extremely useful. An existing PCR-ELISA method based on amplification and detection of the O150 repeat found in the parasites has proven very useful over the last 25 years. An updated real-time qPCR-based method using a mitochondrial DNA target (OvND5) has now been developed that is more sensitive, more species specific, less-time consuming and less costly than the original O150 PCR-ELISA assay. This assay is ready for field evaluation.

## Introduction

Onchocerciasis is an infectious disease caused by the filarial parasite, *Onchocerca volvulus* [1,2]. This parasite is transmitted to humans through the bite of a vector, primarily the blood-seeking blackflies of the *Simulium* genus [3–6]. Onchocerciasis frequently results in severe skin and ocular disease. In the most severe cases, the disease can result in permanently disfigured skin, blindness [7], and is suspected of causing central nervous system pathologies such as epilepsy [8–12].

Community directed treatment with ivermectin (CDTI) is a form of mass drug administration (MDA) that has become the standard of treatment for efforts seeking to eliminate onchocerciasis by interrupting transmission [2,13]. The impact of MDA on transmission of onchocerciasis by blackflies can be verified if there are accurate diagnostic tools to ensure: 1) that the treatment is sufficiently widespread, 2) that interventions are having the intended impact, and 3) that there are no *Simulium spp*. flies containing *O*. *volvulus* infective larvae (stage L3) remaining to transmit the parasite within a geographic area [2,5,9,11,14–20].

The current method recommended by the World Health Organization (WHO) for screening for *O*. *volvulus* infective larvae (L3-stage) in blackflies is a PCR-ELISA method developed 25 years ago that uses the O150 repeat as the molecular target for PCR amplification [21,22]. Although effective at detecting *O*. *volvulus* L3 larvae in pools of blackfly heads, this method would benefit from updating to: 1) simplify the testing procedure, 2) reduce cost, 3) reduce the time required to complete the assay, and 4) use reagents that can be shipped and stored for long periods of time at ambient temperature. [5,23–25].

Several new molecular diagnostic methods have recently been developed to determine the prevalence of *O*. *volvulus* in populations of *Simulium spp*. flies [5,23]. From the suite of choices developed to date, the most sensitive and species-specific tools have been based on nucleic acid amplification tests (NAAT), using either loop-mediated isothermal amplification (LAMP) or real-time quantitative polymerase chain reaction (qPCR) [15,16,21,23,24,26–29]. Each of these methods can determine the presence or absence of the parasite from very small quantities of starting material, and while the LAMP assay has the advantage of being easily adaptable to field settings, it is less sensitive and provides less definitive results than qPCR. Laboratory based real-time qPCR, on the other hand, provides greater sensitivity of detection and allows for a clear and accurate presence/absence read-out of results. New, portable, battery-operated qPCR instruments have recently made it possible for qPCR to become more field friendly [30]. However, the use of these new qPCR systems in the field is still relatively untested. Therefore, for the current optimization project, it was decided to test only laboratory-based qPCR assays that could be run in endemic country laboratories with the potential for high throughput.

With the advent of more widespread qPCR diagnostic testing worldwide (*e*.*g*., for SARS-CoV-2 and HIV), there is increasing accessibility to the required instrumentation across a large number of Lower-and Middle-Income Countries (LMIC). As a byproduct of the SARS-CoV-2 pandemic, there has also been an increased push for the development of stable qPCR reagents, and for the acquisition of real-time PCR instruments all over the globe [31–37]. Such advances have made it possible to adopt qPCR for neglected tropical disease diagnostics at an affordable cost. To develop a standardized, real-time qPCR assay that can be easily adopted for widespread use worldwide, we evaluated several qPCR diagnostic assays developed in the last few years for detecting *O*. *volvulus*. Here, we describe the blind testing of samples across multiple laboratories, to determine the most sensitive, reproducible, reliable, specific, and inhibitor-resistant qPCR assay. Reliability and consistency were evaluated through the testing of high replicate numbers of spiked samples, as well as on field samples from a previously published study [5]. Lastly, we evaluated the use of temperature-stable qPCR reagents in order to reduce logistical costs and improve reliability of the tests under a variety of challenging conditions.

## Methods

All testing was performed at Smith College, unless otherwise noted.

### Genomic DNA isolation from *O*. *volvulus* and *O*. *ochengi*

*Onchocerca volvulus* L3 larvae were isolated at the New York Blood Center (USA) using procedures described previously [38,39]. Genomic DNA (gDNA) from *O*. *volvulus* larvae was extracted using the QIAamp DNA Mini Kit (Qiagen, Hilden, Germany) according to the manufacturer’s protocol and as previously described [40].

*O*. *ochengi* gDNA was extracted from 1 frozen adult female worm using the Wizard Genomic DNA Purification Kit (Promega, Madison, WI, USA) as per the manufacturer’s protocol with the following modifications: the worm was enzymatically digested in Nuclei Lysis Solution containing Proteinase K at 55°C for 2 hrs and the sample was treated at 37°C with RNase, DNase-free, a mixture of ribonucleases produced by Roche (Basel, Switzerland) to remove RNA.

### Preparation of pools of spiked *Simulium* sp. blackfly heads

*Simulium vittatum* cytospecies IS-7 is a non-vector blackfly species, provided by the University of Georgia Black Fly Research and Resource Center. While the use of *S*. *damnosum* would have been optimal, *S*. *vittatum* were chosen for this study because wild-caught *Simulium* species could potentially carry *Onchocerca* species that might impact sensitivity and specificity analyses. Twenty pools of 100 *S*. *vittatum* fly heads were prepared by isolating heads from the bodies using previously described methods [15] with the modification that air-dried flies were placed in a -80°C freezer overnight instead of in liquid nitrogen.

Cryopreserved *O*. *volvulus* L3 larvae were thawed and washed three times in PBS. Individual L3 were rinsed serially in 10, 20 and 100 μL of NET buffer (0.1 M Tris-HCl, pH8; 0.01 M EDTA; and 1M NaCl). The buffer rinses were retained and tested using the NIH O150 qPCR assay for *O*. *volvulus* DNA and the OoR1 qPCR assay to test for *O*. *ochengi* DNA. The testing of buffer rinses was done to ensure that no “free” DNA was present and that there was no contaminating *O*. *ochengi* DNA present prior to conducting any sample preparation for testing. The shipping buffer and first rinse buffer showed sporadic amplification for *O*. *volvulus* DNA, but the second and third rinses did not. None of the buffer rinses were positive for *O*. *ochengi* DNA. Presumably, the *O*. *volvulus* DNA in the initial buffer rinses came from material shed from the L3 larvae. Once rinsed, a single L3 was added to each of 10 pools of 100 blackfly heads. Sample creation occurred at Smith College to ensure that all labs received standardized samples for comparative testing of the qPCR assays. A single L3 larva was used for the creation of each sample because it represents the minimum number of larvae that can be found in a biologically relevant “weak positive” sample encountered under field conditions. Ten additional pools of 100 blackfly heads were not spiked and were used as negative controls.

### DNA isolation from pools of blackfly heads

A modified version of the DNeasy Blood and Tissue kit (Qiagen) was used to extract DNA from pools of blackfly heads. The detailed protocol with modifications and recipes for buffers not provided with the extraction kit is included in S1 Methods. Modifications included a second proteinase K treatment, and additional AW1 buffer rinses. Samples were stored at -20°C prior to being shipped on dry ice to the three participant laboratories.

### Study design for comparison of the candidate qPCR assays

Three different qPCR primer/probe assays designed and intended to detect *O*. *volvulus* with high sensitivity and species-specificity were selected for comparison in this study: two of these assays targeted the *O*. *volvulus* O150 repeat DNA (SC O150, developed at Smith College, and NIH O150, developed at the National Institute of Health), and one targeted the mitochondrial NADH dehydrogenase subunit 5 gene (OvND5). Three *O*. *ochengi*-specific assays (OoND5, OoR1 and OoR5) were also used in this study. OoR1 and OoR5 are newly developed assays that target highly repeated sequences that are specific to *O*. *ochengi* [41]. All of the qPCR probes were labeled with a 5’ FAM fluorophore, an internal ZEN quencher, and a 3’ 3IABkFQ quencher. Probes were synthesized by Integrated DNA Technologies, Inc. (Coralville, IA, USA). Table 1 provides sequences of all primers and probes used in this study and references describing those that have been previously published.

**Table 1:** Primer and probe sequences for all real-time PCR assays used in this study.

Name	Sequence (5’-3’)	Target	Reference
NIH O150 F	CTGATGACCTGTGACCCTAATC	*O*. *volvulus* O150 repeat (141 bp)	this study
NIH O150 R	TCGCCTGTAAATGTGGAA		
NIH O150 PROBE	ACGGGTACATACATTCGAATTGGGTCCC		
SC O150 F	GCCGTGTAAATGTGGAAATTCA	*O*. *volvulus* O150 repeat (214 bp)	this study
SC O150 R1 *	TGATGACCTATGAHCCCTAATHTCA		
SC O150 R2 *	GATTATTAACAGATGACCTATGACATATAATTTCA		
SC O150 PROBE	GGACCCAATTCGAATGTATGTACCCGT		
OvND5 F	GCTATTGGTAGGGGTTTGCAT	*O*. *volvulus* NADH (128 bp)	[16]
OvND5 R	CCACGATAATCCTGTTGACCA		
OvND5 PROBE	TAAGAGGTTATTGTTTATGCAGATGG		
OoND5 F	GCTATTGGTAGGGGTTTGCAT	*O*. *ochengi* NADH (128 bp)	[16]
OoND5 R	CCACGATAATCCTGTTGACCA		
OoND5 PROBE	TAAGAGATTGTTGTTTATGCAGATAGG		
OoR1 F	CGCTAATCACGTGGCTGAT	*O*. *ochengi* (105 bp)	this study
OoR1 R	TCACATGATCAGCACCATTCA		
OoR1 PROBE	CGAGATCACTGCATGCTAAGTCACGT		
OoR5 F	CACTGATCACGTGTCTGTTCAT	*O*. *ochengi* (144 bp)	this study
OoR5 R	GTCAGTGCATGATAGCCAACT		
OoR5 PROBE	CAGTCACGGGATCAGTGCATAGGC		

* Reverse primers R1 and R2 were pooled in equal concentrations

### Initial qPCR primer/probe optimization

Initial qPCR primer/probe optimization was conducted independently at Smith College (SC), the Washington University School of Medicine (WU), and the National Institutes of Health (NIH), using each laboratory’s standard reagents and equipment (see below). DNA template used for testing undiluted unless otherwise noted.

### Smith College reaction conditions and equipment

The SC laboratory used TaqPath ProAmp (TP; ThermoFisher Scientific, Waltham, MA, USA) or FastTaq Universal (FT; ThermoFisher Scientific) for initial testing, since the SC O150 assay had initially been optimized using TP, and all the other assays with FT. Each sample was run in triplicate 7μL reactions (TP) or 10μL reactions (FT). For all TP reactions, a 7μL reaction volume contained 3.5μL of TP, 150nM forward primer, 1000nM reverse primer, 125nM probe, 0.608μL of nuclease-free water, and 2μL of template. Each FT reaction contained 5μL FT, 1μL primer/probe mix (IDT; Integrated DNA Technologies, Inc.), 3μL of nuclease-free water, and 1 μL of template. Smith College used the Applied Biosystems StepOnePlus Real-Time PCR System (ThermoFisher Scientific). Cycling conditions for TP assays had an initial two-minute incubation at 50°C, followed by a 10 min incubation at 95°C and then 40 cycles of 1) 15 sec denaturation at 95°C and 2) one min annealing and extension at 59°C for the SC O150 assay and at 60°C for all other assays. FT assays had an initial 30 sec incubation at 60°C, followed by a one min incubation at 95°C and 40 cycles of 1) one sec denaturation at 95°C and 2) 20 second annealing and extension at 60°C.

### Washington University School of Medicine reaction conditions and equipment

The Washington University laboratory carried out qPCR reactions using the QuantStudio 6Flex Thermocycler (ThermoFisher Scientific) in a 10 μL total reaction volume containing 5 μL TaqMan Fast Advanced Master Mix (ThermoFisher Scientific), 1 μL primer/probe mix (IDT), 1 μL template and 3 μL nuclease-free water. The cycling conditions included a pre-amplification hold at 60°C for 30 sec then denaturation at 95°C for 20 sec, followed by amplification with 40 cycles of 95°C for 1 sec and 60°C for 20 sec. Following amplification, a post-PCR incubation at 60°C for 30 sec occurred. In order to check for any PCR inhibition, the extracted DNA samples were run in parallel reactions with spiked *O*. *volvulus* DNA.

### National Institutes of Health reaction conditions and equipment

The NIH laboratory used TaqMan Fast Advanced Master Mix (ThermoFisher Scientific) for all final qPCR analyses. Each sample was run in triplicate 10 μL reactions on the ViiA 7 Real-Time PCR system (ThermoFisher Scientific). Reactions used the same recipes as those described above for testing at WU. The optimized cycling conditions were 95°C denaturation for 20 secs and then 40 cycles of 1) 1 sec denaturation at 95°C and 2) 20 sec annealing and extension at 60°C.

### Methodological “Targets” specific to project objectives

**Fig 1:**
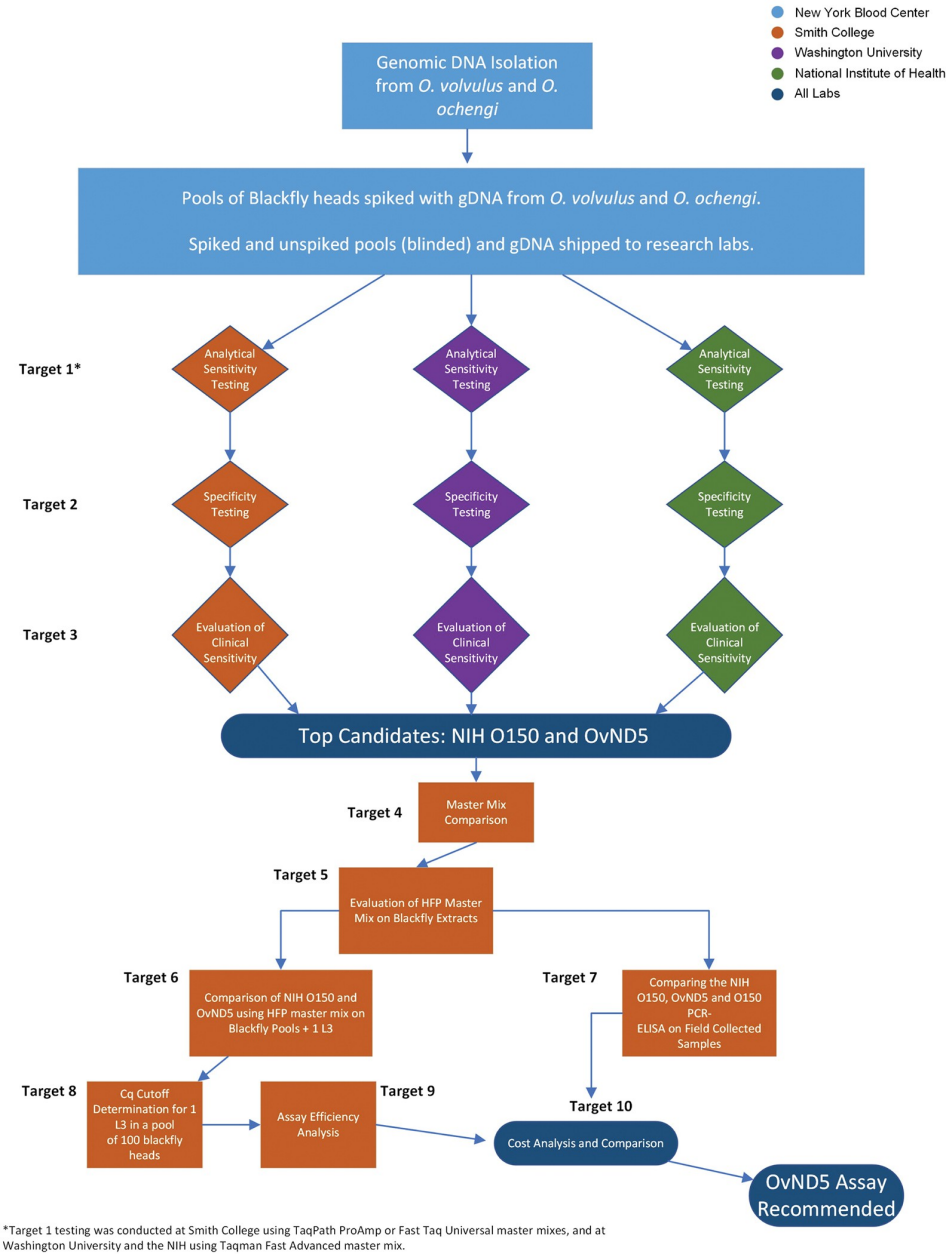
Workflow for the selection of the optimal qPCR assay. Diagram of the steps involved in selecting the optimal *O*. *volvulus* qPCR assay and reagents, including steps to compare the O150 PCR-ELISA assay with the qPCR assay results obtained in this study. Each “Target” described here is identified in the workflow by number.

Our selection criteria are broken down into 10 methodological targets that we utilized to systematically identify an optimal qPCR assay and shelf stable reagents. A diagrammatic representation of the workflow, and the process used to select the most optimal assay and reagents is shown in Fig 1.

#### Target 1: Identification of the analytical limits of detection for the three candidate *O*. *volvulus* qPCR assays

To determine the analytical sensitivity of the three *O*. *volvulus* qPCR assays, *O*. *volvulus* gDNA dilutions at 100 pg/μL, 10 pg/μL, 1 pg/μL, 100 fg/μL, 10 fg/μL, 1 fg/μL, 100 ag/μL, 10 ag/μL, and 1 ag/μL were produced at the New York Blood Center (NYBC) and sent to SC, WU and NIH. These samples were used in each laboratory as the templates for limit of detection testing using the specific conditions described above for each laboratory. This analytical comparison was done on purified O. volvulus gDNA as a pre-screen for significant performance differences between the three assays prior to moving ahead with specificity testing.

#### Target 2: Specificity testing of the three *O*. *volvulus* qPCR assays

Two labs (WU and NIH) provided *O*. *ochengi* DNA to facilitate the specificity testing of each candidate *O*. *volvulus* assay. Specificity tesing against *O*. *onchengi* gDNA occured at concentrations of 1000pg/μL, 100pg/μL, 10pg/μL, 1pg/μL, 0.1pg/μL, and 0.001pg/μL. Previous work has strongly suggested that *O*. *ochengi* is the most closely related *Onchocerca* species to *O*. *volvulus* [42]. For this reason, *O*. *ochengi* DNA was selected as the most appropriate sample type for demonstrating species-specificity of the *O*. *volvulus* assays. These samples were first tested with three *O*. *ochengi*-specific assays OoND5, OoR1 and OoR5 to confirm the identity of the DNA (S1 Table). These DNA samples were then used in each participant laboratory to test the species-specificity of the SC O150, NIH O150 and OvND5 assays, using the reaction conditions and cycling recipes described above.

#### Target 3: Evaluation of clinical sensitivity

Ten pools of 100 blackfly heads were spiked with a single *O*. *volvulus* L3-staged larva, and 10 non-spiked pools were generated. DNA extraction products from the spiked and non-spiked samples were tested with the SC O150, NIH O150 and OvND5 assays in all three laboratories using conditions described above. Samples were also tested with the *O*. *ochengi*-specific OoR1 assay at the SC and NIH laboratories. The *O*. *volvulus* L3 positive/negative status of each sample was blinded to researchers at WU and NIH and to the research associate doing the work at SC.

#### Target 4: Testing six shelf-stable qPCR reagent mixes for *O*. *volvulus* gDNA amplification

Six shelf-stable reagent mixes were selected for testing based on a search for commercially available reagents using the criteria that they not require refrigeration or freezing, and that they utilize a thermostable DNA polymerase. The six reagent mixes were evaluated on their ability to detect *O*. *volvulus* gDNA using the NIH O150 qPCR assay at SC. Mixes included two complete master mixes: one that is stable at room temperature (HFP) and one that is designed to be lyophilized (TC), two polymerases (no master mix) that are designed to be lyophilized (THS and ExTHS), and two polymerases produced as lyophilized "beads" (EB and EBP) (Table 2). Each polymerase/reagent mix was tested at time 0, one week, three weeks and 3 months following storage at ambient temperature (average ~19.5°C). Reagents were tested against *O*. *volvulus* gDNA at a standard concentration (10ng/reaction).

**Table 2:** Shelf-stable reagents/polymerases compared in *O*. *volvulus* DNA detection.

Product	Type	Supplier	Cat #
HOT FIREPol Probe qPCR Mix Plus (HFP)	Temperature stable	Solis Biodyne. Tartu, Estonia	08-14-00020
TaqMan Lyo-ready qPCR Master Mix (TC)	Lyophilizable	Thermo Fisher Scientific. Waltham, MA	C1408SMP
TaKaRa Taq Hot-Start Version (THS)	Lyophilizable	Takara Bio USA, Inc. Mountain View, CA	XA0025
TaKaRa Ex Taq Hot-Start Version (ExTHS)	Lyophilizable	Takara Bio USA, Inc. Mountain View, CA	XA0027
Edvotek PCR beads (EB)	Lyophilized	Edvotek. Bethesda, MD	625
Edvotek PCR beads PLUS (EBP)	Lyophilized	Edvotek. Bethesda, MD	PCR PLUS

The conditions for preparation and use of each of these shelf-stable reagents/polymerases are described in detail in S2 Methods.

#### Target 5: Evaluation of the HFP shelf-stable master mix on blackfly extracts

The NIH O150 assay coupled with the HFP shelf-stable qPCR master mix was also tested at SC against blackfly DNA extracts spiked with *O*. *volvulus* gDNA. These tests were intended to assess the degree to which blackfly extracts inhibited amplification using the shelf-stable master mix. *O*. *volvulus* gDNA was mixed with blackfly DNA extraction product to yield 100 ng of total DNA per sample, including 90 ng of blackfly DNA and 10 ng of *O*. *volvulus* DNA. After mixing, samples were diluted 1:10 in nuclease-free water. Both undiluted and 1:10 dilutions were then used as template in reactions with the HFP qPCR reagents as described above. 10 ng/μL of *O*. *volvulus* gDNA in nuclease-free water was used as a positive control. One μL of 10 ng/μl concentration template DNA was used for each qPCR reaction as described in S2 Methods and as used in the Target 4 methods found above.

#### Target 6: Comparing the NIH O150 and OvND5 qPCR assays using the shelf-stable HFP polymerase to detect *O*. *volvulus* in pools of 100 blackfly heads spiked with a single L3-infective larva

To determine which assay (NIH O150 or OvND5) performed best using the shelf stable HFP reaction mix, 20 pools of 100 *S*. *vittatum* heads were spiked with one *O*. *volvulus* L3 each. All samples then underwent DNA extraction as described above. All 20 extraction products were tested using the NIH O150 and OvND5 qPCR assays coupled with the HFP master mix. All reactions were run in triplicate using 1 μL of extract as template. Due to the presence of inhibitors within the fly extracts all NIH O150 and OvND5 assays produced negative results. 1:20 dilutions of the fly extracts in nuclease-free water were then tested in an effort to overcome this inhibition. The mean quantification cycle (Cq) and standard deviation for the three replicate tests of each of the 20 dilution samples (n = 60) were calculated for both the NIH O150 and OvND5 assays.

#### Target 7: Comparative testing of the O150 PCR-ELISA, the NIH O150 assay, and the OvND5 assay on field samples from Cameroon using the HFP shelf-stable polymerase

Genomic DNA extracts from 129 field samples of blackfly heads previously collected as part of an unrelated study in Cameroon [5] were tested with both the NIH O150 and the OvND5 qPCR assays using the HFP shelf-stable reagents. DNA samples were previously isolated from pools of 100 *Simulium* spp. flies collected using the human landing catch method in the Mbam drainage system in Cameroon. As part of the previous study, all DNA samples were assayed for the presence of *O*. *volvulus* using a LAMP assay and a multiplex PCR assay (OvActin qPCR [5]). In order to evaluate the performance of the NIH O150 and OvND5 assays, these DNA samples were blinded, and then tested using both assays. Results were then compared with those obtained from the O150 PCR-ELISA [27] as well as with the results previously obtained by the original authors using LAMP and OvActin assays. For each 10 μl reaction, the HFP assay contained 2 μL HFP and 1 μL NIH O150 primer/probe mix or OvND5 primer/probe mix (each mix consisted of 500nM of each primer, and 250nM of each probe, and was suspended to a final concentration of 100μM), 6 μl of nuclease-free water and 1 μL of template DNA. The plate was run on a StepOnePlus Real-Time PCR System with an initial two min incubation at 50°C, followed by a 15 min incubation at 95°C and 40 cycles of 1) a 15 sec denaturation step at 95°C and 2) a one min annealing and extension step at 60°C. For the original O150 PCR-ELISA, the assay was carried out on genomic DNA extracted from samples using methods described previously [27].

#### Target 8: Establishing a qPCR cutoff value for the presence of a single *O*. *volvulus* L3 larva in a pool of 100 blackfly heads

The data from Target 6 were used to establish a cutoff value for positive/negative OvND5 qPCR results using the HFP master mix. Recall that 20 pools of 100 S. vittatum heads were spiked with a single L3 larva and 1:20 dilutions of DNA extracts from each pool were tested in triplicate using the OvND5 assay. For each sample, a mean Cq value and standard deviation were determined. The pool with the highest Cq value was used to establish the cutoff by taking the mean Cq value of this sample plus three standard deviations.

#### Target 9: Determining OvND5 qPCR assay efficiency

Assay efficiency was determined using the OvND5 amplicon as template. Ten-fold serial dilutions of the OvND5 amplicon with concentrations ranging from 100 pg/μL to 10 ag/μL were used as template in the qPCR assay. Eleven replicate reactions were run for each tested concentration. The log of the amplicon copy number was plotted against the Cq for each concentration of OvND5 template. A linear regression line was calculated, and the slope of that line was used to calculate the assay efficiency using the formula [43] (S1 Data):

$$E=-1+10^{\left(-\frac{1}{slope}\right)}$$

#### Target 10: Comparing the cost of the OvND5 qPCR assay and the O150 PCR-ELISA

The cost of the OvND5 qPCR assay and the O150 PCR-ELISA assay were estimated based on the inclusion of reagent/supply and labor costs. Labor costs were estimated by using an average of actual salary data from selected endemic laboratories for experienced personnel with some molecular biology expertise. Estimated shipping costs were not included as these vary tremendously from country to country.

## Results

### Target 1: Identification of the analytical limits of detection for the three candidate *O*. *volvulus* qPCR assays

The analytical limits of detection of the three new *O*. *volvulus* qPCR assays were compared at SC, WU, and NIH by testing 10-fold serial dilutions of *O*. *volvulus* genomic DNA ranging from 10 pg/uL to 100 ag/uL. Results from all three laboratories were concordant. The two assays targeting the O150 repeat (NIH O150 and SC O150) had similarly low Cq values but the NIH assay more consistently amplified the lowest concentrations of *O*. *volvulus* genomic DNA. The OvND5 assay was slightly less sensitive, with Cq values about 4 cycles higher than either O150 assay. We determined the limit of detection to be 0.01 pg for the NIH O150 assay and 0.1 pg and for the OvND5 assay (Table 3).

**Table 3:** Limit of detection for the three *O*. *volvulus* qPCR assays.

*Assay*	OvND5			NIH O150			SC O150		
Test Site	WU	SC	NIH	WU	SC	NIH	WU	SC	NIH
**Ov gDNA**									
**10 pg**	29.04	28.28	29.29	24.82	23.95	25.45	25.00	23.71	25.47
**1 pg**	33.05	32.24	33.80	28.88	26.66	29.17	29.50	25.07	29.04
**0.1 pg**	36.86	36.46	35.39	32.40	30.38	31.99	34.80	29.89	36.02
**0.01 pg**	39.36	***	37.11	34.43	34.12	35.06	***	34.09	***
**0.001 pg**	***	***	***	***	***	***	***	***	***

The OvND5, NIH O150 and SC O150 assays were tested in each of three laboratories: Washington University School of Medicine (WU), Smith College (SC), and the National Institutes of Health (NIH). Mean Cq values of three qPCR replicates are reported here

*** indicates no amplification

### Target 2: Specificity testing of the three *O*. *volvulus* qPCR assays

The specificity of each assay was tested using two different *O*. *ochengi* genomic DNA preparations at various concentrations. The NIH O150 and SC O150 assays both amplified *O*. *ochengi* DNA at relatively high concentrations (Table 4). The OvND5 assay did not amplify either of the *O*. *ochengi* genomic DNA samples, even at the highest concentrations (Table 4), and therefore showed the best species-specificity.

**Table 4:** Species-specificity testing for the three *O*. *volvulus* qPCR assays.

*O*. *ochengi* DNA source	Amount of Oo Genomic DNA in the PCR	NIH O150	Assay SC O150	OvND5
**NIH Oo**	**1000 pg**	34.64	36.70	***
**NIH Oo**	**100 pg**	***	***	***
**NIH Oo**	**10 pg**	***	***	***
**NIH Oo**	**1 pg**	***	***	***
**WU Oo**	**1000 pg**	30.95	37.88	***
**WU Oo**	**100 pg**	32.89	***	***
**WU Oo**	**10 pg**	34.31	***	***
**WU Oo**	**1 pg**	***	***	***

Genomic DNA from two different *O*. *ochengi* DNA samples (WU and NIH) were tested to determine the specificity of each qPCR assay. Mean Cq values of three qPCR replicates are reported here.

*** indicates no amplification

### Target 3: Evaluation of clinical sensitivity

Ten pools of 100 blackfly heads spiked with a single *O*. *volvulus* L3 and 10 pools of 100 blackfly heads only were prepared at SC. DNA was extracted, and aliquots were sent to the WU and NIH laboratories. Each laboratory tested these aliquots with the three candidate *O*. *volvulus* qPCR assays, and SC and NIH also tested them with the *O*. *ochengi*-specific OoR1 assay. Results were concordant across all three laboratories with 100% agreement for positive and negative samples for all three assays. Although all three tests gave similar results, the NIH O150 assay again produced a slightly lower mean Cq values than the other two assays (Table 5).

**Table 5:** Mean Cq values of three qPCR assays on spiked blackfly pools.

	Test Site	OvND5			NIH O150			SC O150			NIH OoR1	
		WU	SC	NIH	WU	SC	NIH	WU	SC	NIH	NIH	SC
**Sample 1**	L3 +	26.68	28.53	26.52	24.4	26.49	24.21	26.52	27.16	26.84	***	***
**Sample 2**	L3 -	***	***	***	***	***	***	***	***	***	***	***
**Sample 3**	L3 +	26.54	28.16	28.60	24.68	26.49	24.29	26.52	26.83	26.53	***	***
**Sample 4**	L3 -	***	***	***	***	***	***	***	***	***	***	***
**Sample 5**	L3 -	***	***	***	***	***	***	***	***	***	***	***
**Sample 6**	L3 -	***	***	***	***	***	***	***	***	***	***	***
**Sample 7**	L3 +	27.19	29.04	26.52	25.19	27.47	25.23	27.59	27.48	27.56	***	***
**Sample 8**	L3 -	***	***	***	***	***	***	***	***	***	***	***
**Sample 9**	L3 +	27.41	29.41	26.97	25.9	27.61	25.42	28.23	27.86	28.80	***	***
**Sample 10**	L3 +	27.62	29.54	27.04	26.48	28.49	26.08	28.35	29.06	27.99	***	***
**Sample 11**	L3 +	27.75	29.27	26.94	27.02	28.43	25.94	28.87	29.70	27.86	***	***
**Sample 12**	L3 -	***	***	***	***	***	***	***	***	***	***	***
**Sample 13**	L3 +	28.04	30.06	28.25	25.83	27.71	26.32	28.61	28.58	29.92	***	***
**Sample 14**	L3 -	***	***	***	***	***	***	***	***	***	***	***
**Sample 15**	L3 -	***	***	***	***	***	***	***	***	***	***	***
**Sample 16**	L3 +	28.34	30.27	29.04	25.84	27.79	26.06	28.16	28.62	30.30	***	***
**Sample 17**	L3 -	***	***	***	***	***	***	***	***	***	***	***
**Sample 18**	L3 +	28.04	30.15	29.52	27.63	29.67	28.16	29.80	30.52	31.13	***	***
**Sample 19**	L3 +	28.12	30.46	28.29	27.56	29.78	27.57	29.43	30.33	29.86	***	***
**Sample 20**	L3 -	***	***	***	***	***	***	***	***	***	***	***

Ten pools of 100 blackfly heads with a single *O*. *volvulus* L3 added (L3 +) and ten pools of 100 blackfly heads only (L3 -) were used to test the sensitivity of the OvND5 and the NIH O150 assays in the three laboratories. Additionally, the *O*. *ochengi*-specific assay Oo R1 was tested on the same samples at the NIH and SC laboratories to confirm specificity. Mean Cq values of three qPCR replicates are reported here

*** indicates no amplification.

### Target 4: Testing six shelf-stable qPCR reagent mixes for *O*. *volvulus* gDNA amplification

Six qPCR shelf-stable reaction mixes with DNA polymerase were freshly prepared and tested using the NIH O150 qPCR assay at one week, three weeks and 3 months following storage at ambient temperature. In addition, the HFP was tested at 6 months. The mean Cq of each assay is reported in Table 6. All of the reagents performed well initially, although the ExTHS assay did not amplify at three weeks. The non-lyophilized assay, HFP, produced consistent results over time with simple storage in liquid form at room temperature (and no preparation or lyophilization required). This mix was therefore determined to be the easiest and least expensive qPCR mix to prepare, ship and use. For this reason, the HFP mix was the only reagent tested at 6 months, and it performed as well at 6 months as it did at time 0 (Table 6). As a result, the HFP master mix was selected for use in all further testing.

**Table 6:** Mean Cq of six shelf-stable assays tested over time by the NIH O150 assay.

Product	Time 0	1 week	3 weeks	3 months	6 months
HOT FIREPol Probe qPCR Mix Plus (HFP)	15.90	15.81	15.55	14.91	14.41
TaqMan Lyo-ready qPCR Master Mix (TC)	18.93	18.27	15.90	16.82	ND
TaKaRa Taq Hot-Start Version (THS)	15.56	16.04	16.32	14.81	ND
TaKaRa Ex Taq Hot-Start Version (ExTHS)	15.63	17.37	***	15.34	ND
Edvotek PCR beads (EB)	14.90	11.95	14.50	13.61	ND
Edvotek PCR beads PLUS (EBP)	15.54	14.98	15.25	13.58	ND

Enough of each qPCR assay mix was initially prepared to perform testing at each time point with the same stock aliquot of master mix. All assays except HFP were lyophilized in tubes containing enough reagents for 12 assays. These were rehydrated at each time point and tested using 10 ng of *O*. *volvulus* gDNA as template. The HFP assay was mixed and aliquoted in tubes containing enough reagent for 12 assays. These were kept in the dark at room temperature and tested at each time point using 10 ng of *O*. *volvulus* gDNA as template. Mean Cq values of three qPCR replicates are reported here

*** indicates no amplification.

### Target 5: Evaluation of the HFP shelf-stable master mix on blackfly extracts

The HFP shelf-stable qPCR mixes were next tested on blackfly DNA extracts spiked with 10 ng of *O*. *volvulus* DNA (OvDNA) using the NIH O150 qPCR assay. The undiluted OvDNA+fly extract was not amplifiable with the HFP but was successfully amplified when a 1:10 dilution of the OvDNA+fly extract in nuclease-free water was used. 1:10 sample dilutions of OvDNA without fly extract added were also tested for comparison. With HFP, the diluted OvDNA + fly extract gave a mean Cq value only 3.82 cycles greater than the diluted OvDNA without fly extract (19.78 vs 15.96, Table 7) indicating that HFP is reasonably resistant to inhibition by the blackfly extracts.

**Table 7:** Testing HFP shelf-stable qPCR reagents on blackfly DNA extracts spiked with OvDNA.

	HFP
	Mean Cq
Undiluted Ov DNA + Fly Extract	***
1:10 Diluted Ov DNA + Fly Extract	19.78
1:10 Diluted Ov DNA (no Fly Extract)	15.96

The mean Cq values of three replicate tests per sample are reported

*** indicates no amplification

### Target 6: Comparing the NIH O150 and OvND5 qPCR assays using the shelf-stable HFP polymerase to detect *O*. *volvulus* in pools of 100 blackfly heads spiked with a single L3-infective larva

Twenty pools of 100 blackfly heads, each spiked with a single *O*. *volvulus* L3 larva, were tested with the NIH O150 and OvND5 qPCR assays using the shelf-stable HFP qPCR master mix. qPCR assays for each pool were run in triplicate. As described in Target 5 above, fly pool extracts inhibit qPCR assays. As a result, 1:20 dilutions of each of the extracted DNA samples were tested with the NIH O150 and OvND5 assays. All 20 pool extracts were successfully amplified by the OvND5 assay except Pool 9 (Table 8) while the NIH O150 assay proved more susceptible to inhibitors and failed to produce an amplification product for Pool 9, Pools 8, Pool 11, and Pool 14. The NIH O150 assay also failed to produce a product in two of the three replicates for Pool 3. In addition, the mean Cq for all of the samples that did amplify was 29.0 for OvND5 and 30.3 for NIH O150. The OvND5 results also showed a lower standard deviation (0.62) than the NIH O150 results (1.32).

**Table 8:** Comparison of OvND5 and NIH O150 qPCR performance in detecting one *O*. *volvulus* L3 larva in a pool of 100 *S*. *vittatum* blackfly heads for each of 20 samples.

Spiking experiment: One *O*. *volvulus* L3 larva in 100 Blackfly heads– 20 samples						
	OvND5 Assay			NIH O150 Assay		
Sample	Cq	Mean	SD	Cq	Mean	SD
1	28.90	28.52	0.43	30.47	29.96	0.46
	28.60			29.86		
	28.06			29.56		
2	28.41	28.46	0.10	30.07	30.16	0.08
	28.58			30.20		
	28.40			30.20		
3	28.70	28.89	0.18	***	32.63	N/A
	28.92			***		
	29.06			32.63		
4	29.55	29.24	0.87	30.71	31.11	1.07
	29.90			30.30		
	28.25			32.33		
5	28.71	28.98	0.40	29.08	28.75	0.29
	28.78			28.53		
	29.44			28.64		
6	28.79	29.38	0.85	29.87	29.84	0.21
	28.99			29.62		
	30.36			30.03		
7	28.94	28.86	0.62	31.24	31.48	0.51
	28.21			32.07		
	29.44			31.14		
8	29.11	29.38	0.50	***	N/A	
	29.07			***		
	29.96			***		
9	***	N/A		***	N/A	
	***			***		
	***			***		
10	30.84	30.02	0.72	30.97	30.89	0.18
	29.71			31.01		
	29.49			30.68		
11	28.82	28.60	0.55	***	N/A	
	29.00			***		
	27.97			***		
12	27.79	28.53	0.69	29.63	29.83	0.18
	29.17			29.97		
	28.63			29.88		
13	28.84	29.02	0.18	28.35	28.50	0.18
	29.03			28.45		
	29.21			28.70		
14	28.61	28.64	0.03	***	N/A	
	28.64			***		
	28.66			***		
15	28.68	28.46	0.20	30.04	29.61	0.40
	28.41			29.26		
	28.30			29.54		
16	27.42	27.75	0.34	29.14	28.62	0.49
	28.11			28.16		
	27.73			28.56		
17	29.06	29.78	0.81	28.24	28.25	0.32
	29.63			28.58		
	30.65			27.94		
18	28.69	29.39	0.64	31.99	31.95	0.82
	29.54			32.75		
	29.95			31.11		
19	30.12	30.13	0.60	32.36	31.45	1.20
	30.73			30.09		
	29.53			31.88		
20	30.36	29.77	0.54	31.13	30.99	0.77
	29.29			30.17		
	29.66			31.68		
**Assay Mean**	29.04			30.25		
**SD**	0.62			1.32		

Mean Cq values and standard deviations for 20 samples run in triplicate are reported for each of the assays

*** indicates no amplification

### Target 7: Comparative testing of the O150 PCR-ELISA, the NIH O150 assay, and the OvND5 assay on field samples from Cameroon using the HFP shelf-stable polymerase

DNA isolates from 129 field samples (collected and extracted using the Zymo Research Genomic DNA Tissue Mini-Prep Kit (California, USA) as part of a previously conducted and unrelated study [5]) were each tested in duplicate using the NIH O150 assay and in duplicate using the OvND5 assay coupled with HFP shelf-stable master mix. In addition, 127 of the 129 samples were tested at the University of South Florida with the original O150 PCR ELISA assay for comparison (two samples were not tested by the original O150 because those samples had been exhausted). In the case of differing results between replicates, the samples were re-run in duplicate. The sample was determined to be positive if the retest replicate were both positive, or if one of the replicates was positive and the other negative. If both of the retest replicates were negative, then the sample was determined to be a negative. The results were then shared with the original study’s authors who broke the blinded code (Tables 9 and S2). The NIH O150 and OvND5 assays gave nearly identical results in identifying positive and negative pools. The two tests used in the original study [5], one LAMP and one qPCR, both gave many fewer positive results as did the original O150 PCR-ELISA assay. In addition, while the OvND5 and NIH O150 qPCR assays showed excellent agreement with one another, the other 3 assays did not (S2 Table). These samples were collected as described by Abong, *et al*. [5], following mass drug administration (MDA) with ivermectin in a district in Cameroon: D0 (day zero), D30 (30 days post-treatment), D90 (90 days post-treatment), D180 (180 days post-treatment), and D270 (270 days post-treatment). Unlike the results from the original study and from the results of the original O150 PCR-ELISA, the OvND5 and NIH O150 results show a gradual decrease in positivity over time following MDA.

**Table 9:** Comparison of OvND5 and NIH O150 qPCR results from the 129 pools of *Simulium spp*. flies with results reported by Abong *et al*. [5].

	Positive Detection of OvDNA			
	This Study	Abong *et al*. (2021) [5]		This Study
	NIH O150 and	O150 LAMP	OvActin qPCR	O150 PCR-ELISA
	OvND5 Assays*			
D0	15/15 (100%)	5/15 (33%)	4/15 (26.7%)	3/15 (20.0%)
D30	16/17 (94%)	7/17 (41.2%)	4/17 (23.5%)	6/17 (35.3%)
D90	22/30 (73%)	8/30 (26.7%)	11/30 (36.7%)	7/30 (23.3%)
D180	10/14 (71%)	4/14 (28.6%)	4/14 (28.6%)	6/13 (46.2%)
D270	34/53 (64%)	23/53 (43.4%)	20/53 (37.7%)	20/52 (38.5%)
**Positive Pools**	**97/129 (75%)**	**47/129 (36.4%)**	**43/129 (33.3%)**	**42/127 (33.0%)**

Samples were collected at various time points following Mass Drug Administration (MDA) with of Ivermectin: D0 (day zero), D30 (30 days post-treatment), D90 (90 days post-treatment), D180 (180 days post-treatment), and D270 (270 days post-treatment). The number of positive pools out of the total number pools tested, and the percentage of positive pools is listed for each assay

*Cq values are reported in S2 Table.

Note that all of the samples identified as positive by the O150 LAMP, OvActin qPCR or the original O150 PCR-ELISA were detected as positive (Cq ≤ 33, in >1 replicate PCR) by NIH O150 and the OvND5 assays with the exception of one sample that was positive only using the original O150 PCR-ELISA and one sample that was positive only when tested by the OvActin assay. The new NIH O150 and the OvND5 assays also detected many additional positives at each time point (see S2 Table for complete results for all 129 samples.)

### Target 8: Establishing a qPCR cutoff value for the presence of a single *O*. *volvulus* L3 larva in a pool of 100 blackfly heads

The aim of this target was to identify an appropriate threshold (Cq value) that could be used as a likely indicator of the presence of at least one L3 larva in a pool of 100 blackfly heads using the OvND5 assay. To identify this threshold, we used the results obtained in the exploration of Target 6. In brief, one L3 larva was added to each of 20 pools of 100 blackfly heads. Pools were then extracted and the DNA from each sample was qPCR tested in triplicate. While both the NIH O150 and OvND5 qPCR assays were tested, this analysis was used to develop a Cq threshold for the OvND5 assay only (See Table 8 for Cq values).

The OvND5 assay was demonstrated to be superior to the NIH O150 assay in terms of specificity (Table 4) and sensitivity when testing pools of 100 blackfly heads with a single L3 added (Table 8). Thus, it was next used to establish a threshold value to distinguish a clinically positive qPCR test (defined as resulting from a sample likely to contain at least one L3 larva) from a negative test (defined as resulting from a sample unlikely to contain at least one L3 larva). The mean and standard deviation for Cq values obtained for three replicate tests for each of the 19 samples that amplified by the OvND5 assay (n = 57) were 29.04 and 0.77 respectively. The range for positive Cq values was 27.42 to 30.84. One possible approach to assigning a threshold would be to take the highest value for a single L3 spiked into 100 heads (30.84) and add 3 standard deviations to that to establish a conservative cutoff of 33.2. This cutoff would be conservative, intended to miss as few L3-containing positive samples as possible. Thus, we propose that a Cq value of 33.2 should be considered as a cutoff to discriminate between samples called “positive” and those called “negative”. In other words, any sample with a Cq value less than or equal to 33.2 would be considered positive for the presence of at least one L3-staged larva, while any value greater than 33.2 would be considered negative for the presence of at least 1 L3-staged larva (despite being positive for the presence of *O*. *volvulus* DNA).

### Target 9: Determining OvND5 qPCR assay efficiency

**Fig 2:**
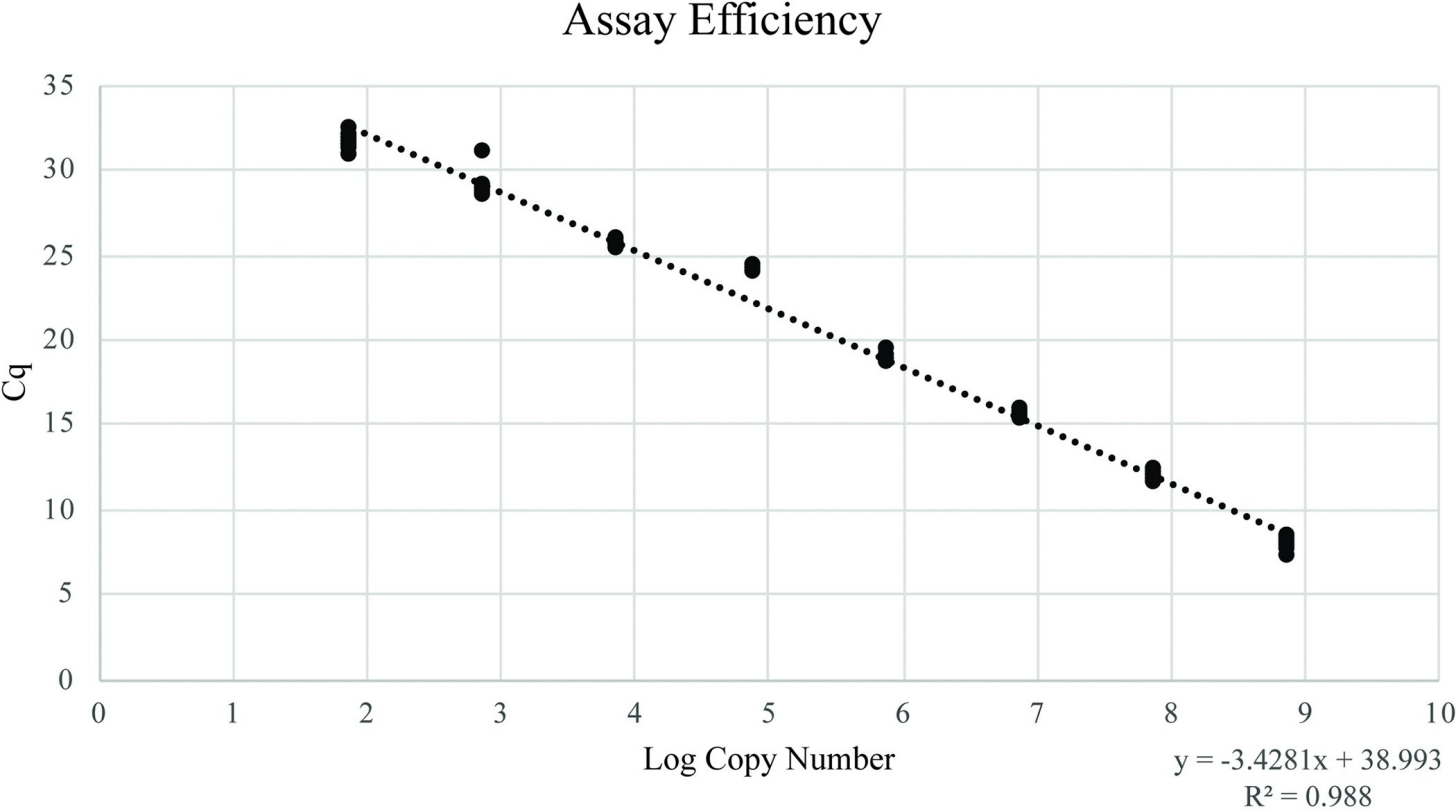
Calculation of assay efficiency. To determine assay efficiency, 10-fold serial dilutions of the OvND5 amplicon were prepared. All dilutions, ranging in concentration from 1ng/μl to 1ag/μl were analyzed in 11 replicate reactions. Mean Cq values and standard deviations were calculated for each concentration of amplicon template (y axis) and these values were plotted against log transformed estimates of copy number for each concentration (x-axis). A slope was then plotted, and reaction efficiency and amplification factor were determined. Estimates of copy number were calculated using the size of the OvND5 amplicon, DNA molecular weight, and the concentration of DNA added.

Using the size of the OvND5 amplicon to estimate the number of copies of OvND5 in successive 10-fold serial dilutions, a linear curve with an R^2^ value of 0.988 was constructed. Linearity was generated by plotting the log transformation of estimated copy numbers. The slope of the line (-3.4281) was used to calculate an assay efficiency of 95.75% and an amplification factor of 1.96 (Fig 2).

### Target 10: Comparing the cost of the OvND5 qPCR assay and the O150 PCR-ELISA

The cost of the OvND5 qPCR assay and the O150 PCR-ELISA assay were estimated based on the inclusion of reagent/supply and labor costs. When reagents, reaction volumes, and labor costs are all included, the cost of the OvND5 assay, per pool tested, is somewhat less than that of the per-pool cost of the O150 PCR-ELISA (see Table 10).

**Table 10:** Cost comparison between the OvND5 qPCR assay and the O150 PCR-ELISA assay.

	O150 PCR-ELISA	OvND5 qPCR
Reagents & Supplies: cost per pool	$7.10	$8.34
Labor (person-hours/plate)	18 hours	4.2 hours
Cost (person-hours per pool)	$1.80	$0.40
Total Cost Per Pool	$8.90	$8.74

Assays were compared based on cost per pool of flies, building in supply and labor costs (estimated at $9.00 US per hour). It should be noted that the OvND5 qPCR has many fewer steps and is less labor intensive than the O150 PCR-ELISA (18 hours vs 4.2 hours).

## Discussion

The global elimination of onchocerciasis represents a massive technical and political challenge involving many governments, agencies, partners, and countries [44–48]. Defining success in the elimination of onchocerciasis transmission is based, in part, on assessing the absence of parasites in the heads of the transmitting *Simulium* spp. blackflies. Originally, this assessment involved the laborious, and less sensitive, manual dissection of the flies–a technique that was time-consuming and required specific parasitological expertise [27]. Since the infectious L3 stage of the parasites migrates to the heads of the fly prior to transmission to a human host, molecular techniques were developed to ascertain the presence/absence of parasites in the heads of blackflies. The original PCR-ELISA technique based on detecting the O150 repeat found in *O*. *volvulus* and other *Onchocerca* species was an important advance in vector screening [21]. This method has provided a great deal of information essential for evaluating the progress of onchocerciasis elimination programs, and the success of many such programs would not have been possible without this important tool.

The effective programmatic use of molecular assays requires the presence of appropriately equipped laboratories and trained technical staff capable of processing large numbers of field samples in a timely fashion. Collection of accurate data on the status of infection in an endemic area is key to critical managerial decisions that national programs must make, and this is especially relevant as their CDTI/MDA efforts reach the point of requiring a decision regarding whether to stop treatment or continue with further MDA. The approach to making these important decisions is currently based on screening of blackfly heads by PCR-ELISA, supported by serology that is limited by suboptimal techniques for identifying the Ov16 antibody in children who were born following the onset of MDA. Given the absence to date of any optimal point-of-collection NAAT diagnostic for screening blackly heads (which arguably would be preferred), it has been necessary to utilize the O150 PCR-ELISA assay in established, technically capable laboratories for programmatic decision making [49]. While efforts have been made to support laboratories across Africa and Yemen to respond to the ever-escalating number of field samples that must be tested, it is clear that streamlining and reducing the cost of the molecular assay is essential to ensuring the future success of elimination programs. In addition, as positivity rates in vectors decrease and national programs move closer toward stopping treatment, the sensitivity and specificity of the assays used to screen pools of blackflies becomes more critical in demonstrating the successful interruption of transmission. Thus, the goal of the present study was to design a qPCR assay with the characteristics of shorter testing time, reduced cost, improved specificity, and reduced chances of PCR contamination.

In the first part of this study (Targets 1, 2, and 3), we found that two new O150 based qPCR assays (NIH O150 and SC O150) and the OvND5 assay were all excellent at detecting picogram quantities of purified *O*. *volvulus* genomic DNA as well as picogram quantities of *O*. *volvulus* gDNA added to pools of 100 blackfly heads. While all three qPCR assays were very sensitive, the OvND5 assay had the advantage of having improved specificity (i.e. no detection of *O*. *ochengi*).

In the second part of the study (Targets 4, 5, and 6), we focused on finding a solution for a major practical issue associated with carrying out qPCR assays in endemic countries; namely, the high cost of using reagents that must be shipped and stored at -20°C (or colder). Shipment delays in customs, freezer breakdowns, or erratic power availability in endemic laboratories are all problems that can result in the loss of expensive reagents, and arguably even more critically, significant delays in operations while waiting for replacement reagents to arrive. The availability of new shelf-stable polymerases and reagents that can be shipped at room temperature has now provided an opportunity to reduce shipping and storage costs associated with PCR assays, to eliminate failure-based waste, and to reduce critical time delays. Thus, for these studies, we evaluated six different DNA polymerases and qPCR master mixes that can be shipped and stored at room temperature. While all of these qPCR polymerases/master mixes performed well, by far the easiest to use was HFP (HOT FIREPol Probe qPCR Mis Plus produced by Solis BioDyne). This mix was easiest to use because it comes in a ready-to-use liquid form with dNTPs, requiring no manipulation (lyophilization) or resuspension in buffer. All other polymerases/mixes tested required such procedures. The HFP master mix is also stable when stored at room temperature. In our hands, this master mix was just as effective after six months of storage at room temperature as it was when first received from the manufacturer. Not having to lyophilize reagents means less opportunity to introduce inconsistencies which are often observed with lyophilized products. The elimination of cold shipment and storage needs also represents a large cost-savings. When the reagents arrive at their destination, cold storage is still recommended, but the stability of the HFP polymerase/master mix means there is little concern should a freezer fail or power interruption occur. The results of this phase of the study also demonstrated that the OvND5 assay exhibited improved sensitivity compared to the O150 assays when using HFP on pools of 100 blackfly heads with a single L3 added. This improved sensitivity was likely the result of superior inhibitor-resistance of the OvND5 assay when coupled with the HFP master mix.

Of note, we recognize the increased analytical sensitivity of the NIH O150 assay based on the LOD experiments, and our data demonstrate Cq values for the OvND5 assay that were consistently higher than the corresponding Cq values for the NIH O150 when testing the same set of samples. When we replicated near real-world conditions (20 pools of flies spiked with a single L3 larva) the NIH O150 assay underperformed in comparison to the OvND5, with a higher mean Cq value and an increased standard deviation (NIH O150 mean = 30.14, SD = 1.3; OvND5 mean = 29.04, SD 0.75). A higher rate of false negative results was also seen using the NIH O150 assay, and further dilution of the samples did not improve the results (S3 Table). For these reasons, as clinical performance is ultimately more relevant than analytical results, we selected the OvND5 assay as our recommended diagnostic option.

Finally, in the third part of the study (Targets 7, 8, and 9), the original O150 PCR-ELISA assay, the NIH O150 assay and the OvND5 assay were comparatively tested against DNA extracts from blackfly pools collected as part of an earlier study in Cameroon [5]. In this study, a LAMP assay and a different qPCR assay (OvActin qPCR) were used to detect the presence or absence of *O*. *volvulus* DNA in the samples. The results of this comparison showed that the OvND5 and the NIH O150 assays using HFP were in near-perfect agreement, while the LAMP and OvActin assays produced considerably fewer positive results. Of note, all of the positive results detected by the original O150 PCR-ELISA, the O150 LAMP and the OvActin assays, were detected by the OvND5 and the NIH O150 qPCR assays. In light of the aforementioned performance characteristics and given the strong benefits of utilizing an ambient temperature-stable polymerase, we believe that the OvND5 assay using the HFP polymerase/master mix provides the ideal qPCR assay for detecting *O*. *volvulus* in blackflies. Its sensitivity, species-specificity, cost effectiveness, and logistical advantages make it superior to all other assays tested.

Using the data collected from 19 out of 20 pools of 100 blackfly heads each spiked with a single L3 larva and tested by qPCR in triplicate, an effort was made to determine a threshold for biologically relevant positivity using the OvND5 assay. Selecting the ideal cutoff value for such positives in any molecular assay is difficult, and qPCR assays are no exception. Some labs identify any Cq value below 40 as a positive, while others use 38 as a cutoff. While we believe that any positive result is indicative of the presence of *O*. *volvulus* target DNA, we proposed consideration for a cutoff Cq value of 33.2 because this is 3 standard deviations above the highest Cq obtained in our spiking experiments containing many replicate pools of 100 blackfly heads spiked with a single L3 larva. Such pools theoretically represent the minimum worm burden required for a sample to represent an infection risk. Since the standard deviation was relatively low (0.79) this meant that 2.37 was added to 30.84 to give a Cq cutoff of 33.2. As work goes forward and additional test results become available, this cutoff value should continue to be evaluated and reconsidered and such evaluation will be particularly important as testing expands to new laboratories utilizing different qPCR instruments.

Additional tests on endemic blackfly vectors that are the species most likely to transmit onchocerciasis will help to evaluate the extraction methods and resistance of the assay to PCR inhibitors. One of the 20 pools failed to amplify, even with additional dilutions of the sample (S3 Table). It’s not clear whether this result is due to inhibition, error, or some other factor. Future experiments to replicate the result would help to elucidate this.

To assess the implications of assay choice on study expense and logistical difficulty, the relative cost and complexity of the OvND5 qPCR assay and the O150 PCR-ELISA were compared. Although improvements to the O150 PCR-ELISA have resulted in the utilization of lyophilized reagents, the assay is still marginally more expensive due to the higher number of person-hours required to complete the more complex protocol. The OvND5 assay has fewer steps, takes less time to complete, and has reduced contamination risks because it eliminates the handling of post-PCR products as it is conducted in a sealed 96 well plate. This assay also demonstrated improved sensitivity when compared with the O150 PCR-ELISA.

In this multi-laboratory collaborative study, we have compared different qPCR targets in *O*. *volvulus*, different primers and probes, and different PCR polymerase/master mixes in order to develop an agreed upon protocol for screening pooled blackflies that is faster, more streamlined, more sensitive, more species-specific and more cost-effective than the currently available O150 PCR-ELISA assay. The OvND5 qPCR assay, with all reagents delivered to the testing laboratory at ambient temperature, will minimize logistical challenges while avoiding the high costs of cold chain shipping as well as the potentially expensive losses that result when logistics-related delays occur. In addition, a qPCR assay performed in a sealed plate has the advantage of greatly reducing the chances of sample-to-sample contamination and general laboratory contamination with concentrated PCR amplification products. This optimized and standardized OvND5 qPCR protocol for the monitoring of *O*. *volvulus* in vector blackflies in endemic countries will facilitate the more-timely processing of samples, more reliable results, and will reduce delays in programmatic decision-making. With standardized approaches and stable reagents available, the network of laboratories supporting molecular xenomonitoring efforts for onchocerciasis can be enlarged, providing governments and agencies responsible for elimination programs with the reliable data needed to aid in stop-treatment decision-making and post-MDA surveillance.

## Supporting information

S1 Methods Detailed protocols for DNA isolation from pools of blackfly heads. (PDF)

S2 Methods Detailed protocol for preparing and testing shelf-stable qPCR reagents on genomic *O*. *volvulus* gDNA. (PDF)

S1 Table Cq values confirmatory assay to test for species specificity of the O. ochengi-specific assays: OoND5 and OoR1 and OoR5. (PDF)

S2 Table Comparison of results obtained for detection of O. volvulus in 129 Cameroon samples. (PDF)

S3 Table Comparison of OvND5 and NIH O150 qPCR performance in detecting one *O*. *volvulus* L3 larva spiked into a pool of 100 *Simulium vittatum* blackfly heads at dilutions of 1:20 and 1:50. (PDF)

S1 Data Efficiency Assay Cq values, calculations, plot and data. (XLSX)
