# Supplementary material for: Optimized strategy for real-time qPCR detection of Onchocerca volvulus DNA in pooled Simulium sp. blackfly vectors
Source: PLoS Negl Trop Dis. 2023 Dec 14;17(12):e0011815. doi: 10.1371/journal.pntd.0011815 (PMC10754622; doi:10.1371/journal.pntd.0011815)
Supplement: S1 Methods — (PDF) [file pntd.0011815.s001.pdf]

S1 Methods. Detailed protocol for DNA Extraction from Pooled *Simulium damnosum* heads

Notes:

1. This protocol uses the Qiagen DNeasy Blood and Tissue kit (Qiagen cat # 69506 for 250 column kit) with a modified method that requires some additional reagents (Proteinase K, Qiagen cat # 19133 and Buffer AW1, Qiagen cat # 19081) to be ordered.
2. Ethanol must be added to Qiagen Buffers AW1 and AW2 prior to use. Add ethanol according to instructions found on the buffer bottles that come in the kit.
3. Preheat TempBloc incubators to 56°C and 70°C at the beginning of each work day.
4. 95% ethanol and 1X PBS (Fisher Scientific cat # BP2944100) needs to be provided by the user.
5. When opening tubes throughout the procedure, use a microcentrifuge tube opener to prevent cross-contamination of samples.
6. Change gloves frequently, to avoid cross-contamination of samples.
7. Two protocols are included here, one using a Qiagen Tissuelyser II to macerate samples, and one using plastic pestles. Note that the protocol using the Tissuelyser II instrument is highly recommended and preferred.

Extractions supplies needed:

1.7 mL microcentrifuge tubes (Phenix Research Products cat # MAX-715)

15 mL tubes (USA Scientific cat #5618-8271)

Qiagen DNeasy column/collection tube (component of DNeasy Blood and Tissue Kit, Qiagen cat # 69506)

Additional collection tubes (component of DNeasy Blood and Tissue Kit)

1X PBS (Fisher Scientific cat # BP2944100)

Proteinase K (component of DNeasy Blood and Tissue Kit, but extra must be purchased, Qiagen cat # 19133)

Buffer AL (component of DNeasy Blood and Tissue Kit)

95% molecular biology grade Ethanol

Buffer AW1 (component of DNeasy Blood and Tissue Kit, but extra must be purchased, Qiagen cat # 19081)

Buffer AW2 (component of DNeasy Blood and Tissue Kit)

Buffer AE (component of DNeasy Blood and Tissue Kit)

Microcentrifuge tube openers (USA Scientific cat # 1400-1508)

Vortex

Microcentrifuge capable of reaching 16,000 xg

Micropipettors and tips (p1000, p200, p10): We recommend:

1-10 µL Gilson Single Channel Pipette (Gilson cat # FA10002M)

20-20 µL Gilson Single Channel Pipette (Gilson cat # FA10003M)

20-200 µL Gilson Single Channel Pipette (Gilson cat # FA10005M)

100-1000 µL Gilson Single Channel Pipette (Gilson cat # FA10006M)

Pestles for microcentrifuge tubes (Fisher Scientific cat #12-141-363)

Extraction control *B. atrophaeus* stock dilution (Thermo Fisher A39180)

For main protocol (not needed for alternate protocol below):

Tissuelyser II (Qiagen Cat No./ID: 85300)

2.0mL Eppendorf tubes

Zinc-plated 0.177 caliber BB (4.5mm) Steel Airgun Shot

Preferred Extraction Protocol, using the Qiagen TissueLyser II:

1. Add 1 autoclaved zinc-plated BB to each 2mL microcentrifuge tube containing up to 100 black fly heads
2. Using a P200 with sterile filter tips for all samples, add 180µl 1X PBS. Be careful not to touch the tip to any of the tubes during this addition. Change tip as needed, if it touches anything.
3. Pipette 5µl of the stock solution of the extraction control, *B. atrophaeus* to each sample.
4. Securely close all tubes and place all tubes in the TissueLyser II. Macerate samples for 10 minutes at a frequency setting of 30.0. It is important that labels be placed on the sides of each sample tube prior to using the TissueLyser II as labels are removed from lids by the vigorous shaking which occurs during this step.
5. Centrifuge all sample <u>briefly</u> to collect sludge away from the lid of the tube and to prevent cross-contamination in subsequent steps.
6. Using a microcentrifuge tube opener, open all sample tubes.
7. Using a P20 pipette with a new sterile filter tip for each sample, add 20 µL of Qiagen Proteinase K to each tube. Then, using a P200 pipette with a new sterile filter tip for each sample, add 200 µL of Buffer AL to each sample tube.
8. Following buffer addition, immediately mix each sample by vortexing the tube just long enough to resuspend any pelleted material (about 3 seconds).
9. Incubate all samples at 70°C for 10 minutes in a TempBlock incubator.
10. Centrifuge all tubes briefly to remove any condensation/liquid from the cap area to prevent cross-contamination when opening tubes in subsequent steps.
11. Using a microcentrifuge tube opener, open all tubes and add an additional 20 µL of Qiagen Proteinase K to each sample. Use a new P20 sterile filter tip for each sample.

12. Mix all samples by vortexing the tubes just long enough to resuspend any pelleted material (about 3 seconds).
13. Incubate all tubes at 56°C for 1 hour in a TempBlock incubator.
14. Centrifuge all tubes at maximum speed (16,000 x g or greater) for 5 minutes to pellet debris.
15. While samples are centrifuging, using a P1000 pipette and the same sterile, filtered P1000 pipette tip for all tubes, add 200 µL of 95% ethanol to a series of new 1.7 mL microfuge tubes. One tube of ethanol should be prepared for every sample tube.
16. While samples are centrifuging, label a series of DNeasy spin columns. One spin column should be prepared for every sample tube.
17. Following centrifugation, using a P1000 pipette and a new sterile filtered P1000 pipette tip for each sample, transfer the supernatant from each sample tube to a 95% ethanol-containing tube prepared in step number 14. Using the same pipette tip that was used to transfer each sample, gently pipet the sample-ethanol mixture up and down to mix, then apply the entire sample to a DNeasy spin column. (Note: Be sure to use a microcentrifuge tube opener to open all sample tubes.)
18. Centrifuge all samples at 8,000 x g for 1 minute.
19. Dispose of the flow-through from each sample into a waste collection beaker and transfer each DNeasy Spin column to a new waste collection tube. Discard the first waste collection tube.
20. Using a P1000 pipette and a new, sterile P1000 filtered pipette tip for each sample, add 500 µL of Buffer AW1 to each DNeasy Spin column.
21. Centrifuge all DNeasy Spin columns at 8,000 x g for 1 minute.
22. Following centrifugation, dispose of the flow-through from each sample into a waste collection beaker, gently blot each collection tube by tapping it a few times on an absorbent paper towel,

then return each column to the same collection tube. (Note: To prevent sample cross-contamination, be certain that each collection tube is blotted in a clean, unused position on the absorbent towel. Do not blot multiple tubes in the same location.)

23. Using a P1000 pipette and a new, sterile P1000 filtered pipette tip for each sample, add another 500 µL of Buffer AW1 to each DNeasy Spin column.
24. Centrifuge all DNeasy Spin columns at 8,000 x g for 1 minute.
25. Following centrifugation, dispose of the flow through from each sample into the waste collection beaker, discard the waste collection tube, and move the column to a new waste collection tube.
26. Using a P1000 pipette and a new, sterile, filtered P1000 pipette tip for each sample, add 500 µL of Buffer AW2 to each DNeasy Spin column.
27. Centrifuge all columns at maximum speed for 3 minutes in the microcentrifuge.
28. Following centrifugation, dispose of the flow through from each sample into a waste collection beaker, gently blot each collection tube by tapping it a few times on an absorbent paper towel, then return each column to the same collection tube. (Note: To prevent sample cross-contamination, be certain that each collection tube is blotted in a clean, unused position on the absorbent towel. Do not blot multiple tubes in the same location.)
29. Centrifuge all DNeasy Spin columns for an additional 3 minutes at maximum speed to ensure that all residual ethanol from the wash buffers is removed from the column membranes.
30. Transfer each DNeasy Spin column to a new, labeled 1.7 mL microcentrifuge tube.
31. Using a P200 pipette with a new, sterile filter tip for each sample, add 125 µL of elution buffer AE to each DNeasy Spin column. Allow the buffer to sit on the column membrane at room temperature for <u>at least 2 minutes</u>. (Note: When adding Buffer AE to each DNeasy Spin column be careful not to touch your pipette tip to the column. Rather, allow the buffer to fall into the

center of the column by carefully releasing the buffer from the pipette tip from a position directly above the center of the column bed. Poking the columns surface with the pipette tip can damage the column bed and result in compromised DNA recovery.)

32. Centrifuge all DNeasy Spin columns at 8,000 x g for 2 minutes.
33. Following centrifugation, using a tube opener, open each DNeasy spin column.
34. Carefully working with one DNeasy Spin column at a time, carefully remove the column from the collection tube and with a P200 pipette and a new, sterile filtered P200 pipette tip for each sample, aspirate the flow-through from the collection tube. Carefully reload the entire volume of this aspirated liquid (which spun through the column) back onto the center of the column membrane.
35. Following the addition of the "flow-through" back to the column for each sample, allow all columns to sit at room temperature for <u>at least 2 additional minutes</u>.
36. Centrifuge all DNeasy Spin columns at 10,000 x g for an additional 2 minutes. The resulting "flow-through" is your extracted black fly DNA sample.
37. Continue with qPCR testing as soon as possible after the extraction. Samples should be stored at 4°C until qPCR results are obtained (assuming testing will happen within 5-7 days)***. Samples should then be moved to -20°C for long-term storage.

***If samples have been stored at 4°C before performing qPCR, make sure that you briefly spin all DNA sample extract tubes before opening their lids to move any collected condensation away from tube lids.

<u>Alternative Protocol, not using the Qiagen TissueLyser II (replaces steps 1-3 in the above protocol)</u>

1. Beginning with pools of fly heads in 1.7 µL microcentrifuge tubes, carefully open all sample tubes with a microcentrifuge tube opener.
2. Using a P200 pipette and the same sterile filter tip for all samples, add 90 µL 1X PBS to each sample tube. Be careful not to touch the tip to any of the tubes during this addition. Discard and replace your tip if it contacts a sample tube during PBS addition.
3. Using a clean pestle for each sample, macerate samples for 60 seconds. Following maceration, rinse the pestle into your sample tube by gently pipetting an additional 90 µL 1X PBS along the length of the pestle.

Continue to step 4 of the main protocol

Recipes

Phosphate Buffered Saline (PBS)

Reagent	Amount	Final concentration
NaCl	8 g	137 mM
KCl	0.2 g	2.7 mM
Na_2HPO_4	1.44 g	10 mM
KH_2PO_4	0.24 g	1.8 mM

To prepare 1 L of 1× PBS, dissolve the reagents listed above in 800 mL of nuclease-free H_2O. Adjust the pH to 7.4 with HCl, and then add nuclease-free H_2O to 1 L. Autoclave your 1 x PBS stock for 20 minutes on the liquid cycle or filter sterilize the solution. Following sterilization, create 5 mL aliquots of 1 x PBS from your sterilized stock. PBS aliquots may be stored at room temperature indefinitely.

B. atrophaeus extraction control stock solution

- The sample is shipped in 3 freeze-dried ‘TaqMan™ Universal Extraction Control Organism’ glass vials.
- Remove rubber stopper from one vial, taking care to not release the powder, and add 200µl 1x PBS to the vial. Ensure all of the powder is dissolved.
- Pipette the 200µl of resuspended *B. atrophaeus* to a 1.7mL tube.
- Using a fresh tip, pipette a second 200µl of 1x PBS to the vial, and rinse the vial to get any remaining cells, and add to the 1.7mL tube.
- Add 600µl additional 1x PBS to the 1.7mL tube and mix (final volume is 1mL)
- The “stock” solution is a 1:100 dilution of the resuspended cells. Create the “stock” solution by pipetting 990µl of water into fresh tubes, and adding 10µl of the cell suspension to each and vortexing. Store the “stock” solution at -20°C for use as an extraction control.
