# Supplementary material for: Optimized strategy for real-time qPCR detection of Onchocerca volvulus DNA in pooled Simulium sp. blackfly vectors
Source: PLoS Negl Trop Dis. 2023 Dec 14;17(12):e0011815. doi: 10.1371/journal.pntd.0011815 (PMC10754622; doi:10.1371/journal.pntd.0011815)
Supplement: S2 Methods — (PDF) [file pntd.0011815.s002.pdf]

Detailed protocol for preparing and testing shelf-stable qPCR reagents on genomic *O. volvulus* gDNA

Hot FirePol Reaction Mix

For each 10 µl reaction, the Hot FirePol (HFP) assay contained 2 µL HFP and 1 µL NIH O-150 primer/probe and 6 µl of nuclease-free water. Aliquots with enough mix for 12 reactions were produced, sealed with parafilm, and stored in the dark at room temperature for one week, three weeks, three months and six months. To process, 9 µL of the mix was added to each well of a 96-well Semi-skirt 0.1ml PCR plate (USA Scientific), 1 µL of *O. volvulus* genomic DNA template (10 ng) was added, and the plate was run on a StepOnePlus Real-Time PCR System with an initial two min incubation at 50°C, followed by a 15 min incubation at 95°C and 40 cycles of 1) a 15 sec denaturation step at 95°C and 2) a one min annealing and extension step at 60°C.

TaqMan Lyo-ready qPCR Master Mix

For each 10 ul reaction, the TaqMan Lyo-ready qPCR Master Mix (TC) assay contained 2 µL TC, 1 µL NIH O-150 primer/probe, and 0.1 µL 50 uM ROX. A batch with enough for 50 reactions was prepared containing 100 µL TC, 50 µL NIH O-150 primer/probe, and 5 µL 50 uM ROX. This was divided into four 38.75 uL volumes. To one tube, 86.25 µL of nuclease-free water was added, mixed well, and briefly centrifuged. To each well in a row on a 96 well plate, 9 µL of the mix and 1 µL of *O. volvulus* genomic DNA template (10 ng) was added. The plate was run on a StepOnePlus™ Real-Time PCR System with an initial two min incubation at 50°C, followed by a 10 min incubation at 95°C and 40 cycles of 1) a 15 sec denaturation step at 95°C and 2) a one min annealing and extension step at 60°C. This was the "time 0" assay. The remaining tubes of PCR mix were lyophilized as described below, sealed with parafilm and stored in a desiccator in the

dark at room temperature for testing at one week, three weeks, and three months. To process each of these stored tubes of PCR mix, each tube was rehydrated with 125 µL nuclease-free water, allowed to sit on ice for five min, mixed well, and briefly centrifuged. To each well in a row on a 96 well plate, 9 µL of the mix and 1 µL of *O. volvulus* genomic DNA template (10 ng) were added. The plate was run on a StepOnePlus™ Real-Time PCR System with an initial two min incubation at 50°C, followed by a 10 min incubation at 95°C and 40 cycles of 1) a 15 sec denaturation step at 95°C and 2) a one min annealing and extension step at 60°C.

<u>TaKaRa Lyo-ready Taq Hot Start Reaction Mix</u>

For each 10 µl reaction, the TaKaRa Lyo-ready Taq Hot Start (THS) and the TaKaRa Lyo-ready EX Taq Hot Start (ExTHS) assay contained 0.048 µL THS or ExTHS, 1 µL NIH O-150 primer/probe, 0.1 µL 50 µM ROX, 0.6 µL dNTP, 2 µL 5X PCR buffer, and 1.6 µL $MgCl_2$. A batch with enough for 50 reactions was produced, containing 2.4 µL THS or ExTHS, 50 µL NIH O-150 primer/probe, 5 µL 50 µM ROX, and 30 µL dNTP mix. This was divided into 4 aliquots of 21.85 µL. To one tube, 25 µL of 5X PCR buffer, 20 µL 50 mM $MgCl_2$, and 58.15 µL of nuclease-free water were added, mixed well, and centrifuged briefly. To each well in a row on a 96 well plate, 9 µL of the mix and 1 µL of *O. volvulus* genomic DNA template (10 ng) were added, and the plate was run on a StepOnePlus Real-Time PCR System with an initial two min incubation at 50°C, followed by a 10 min incubation at 95°C and 40 cycles of 1) a 15 sec denaturation step at 95°C and 2) a one min annealing and extension step at 60°C. This was the "time 0" assay. The remaining 3 tubes were lyophilized as described below, sealed with parafilm and stored in a desiccator in the dark at room temperature for one week, three weeks, and three months. To process the lyophilized tubes, they were rehydrated with 80 µl nuclease-free water, 25 µl 5X PCR

buffer, and 20 µl $MgCl_2$, allowed to sit on ice for five min, mixed well, and centrifuged briefly. To each well in a row on a 96 well plate, 9 µL of the mix and 1 µL of *O. volvulus* genomic DNA template (10 ng) was added, The plate was run on the StepOnePlus™ Real-Time PCR System with an initial two min incubation at 50°C, followed by a 10 min incubation at 95°C and 40 cycles of 1) a 15 sec denaturation step at 95°C and 2) a one min annealing and extension step at 60°C.

<u>PCR EdvoBeads</u>

For each 10 µl reaction, the PCR EdvoBeads (EB) and the PCR EdvoBeads PLUS (EBP) assays contained 1 µL NIH O-150 primer/probe, 0.1 µL 50 µM ROX, 1.6 µL $MgCl_2$ and 7.3 µl nuclease-free water. Four aliquots with enough mix for five reactions were produced, with 5 µL NIH O-150 primer/probe, 0.5 µL 50 µM ROX, 8 µL 50 mM $MgCl_2$ and 36.5 µl nuclease-free water. To two tubes, 2 EB beads and 29.5 µL of nuclease-free water or 2 EBP beads and 29.5 µL of nuclease-free water were added, mixed well, and briefly centrifuged. To each well in a row of a 96 well plate, 9 µL of these mixes and 1 µL of *O. volvulus* genomic DNA template (10 ng) were added and the plate was run on a StepOnePlus™ Real-Time PCR System with an initial two min incubation at 50°C, followed by a 10 min incubation at 95°C and 40 cycles of 1) a 15 sec denaturation step at 95°C and 2) a one min annealing and extension step at 60°C. These were the "time 0" assays. The remaining three tubes were lyophilized as described below. To each tube of lyophilized mix, 2 EB or EBP beads were added. The tubes were sealed with parafilm and stored in a desiccator in the dark at room temperature for one week, three weeks, three months and six months. To process, the tube was rehydrated with 45 µl nuclease-free water, allowed to sit on ice for five min, mixed well, and centrifuged briefly. To each well in a row on a 96 well plate, 9 µL of the mix and 1 µL of *O. volvulus* genomic DNA template (10 ng) were added, and the plate was

run on the StepOnePlus Real-Time PCR System with an initial two min incubation at 50°C, followed by a 10 min incubation at 95°C and 40 cycles of 1) a 15 sec denaturation step at 95°C and 2) a one min annealing and extension step at 60°C.

Preparation for Lyophilizable Mixes

Each lyophilizable assay mix (TC, THS, ExTHS) was prepared as described above, with enough material for 12 reactions divided into each of five 1.5 mL microcentrifuge tubes. To each tube, an equal amount of 2X lyophilization reagent (a proprietary excipient mix, OPS Diagnostics, Lebanon NJ) was added. These tubes were flash-frozen in liquid nitrogen and placed into a lyophilizer in 50 mL conical tubes covered with absorbent tissues. Samples were dried at -80°C for 20 hours, at which time they were removed from the lyophilizer, sealed with parafilm, and stored in the dark in a foil-wrapped desiccator.
