# Supplementary material for: Optimized strategy for real-time qPCR detection of Onchocerca volvulus DNA in pooled Simulium sp. blackfly vectors
Source: PLoS Negl Trop Dis. 2023 Dec 14;17(12):e0011815. doi: 10.1371/journal.pntd.0011815 (PMC10754622; doi:10.1371/journal.pntd.0011815)
Supplement: S1 Table — (PDF) [file pntd.0011815.s003.pdf]

S1 Table: Cq values for confirmatory assay to test for species specificity of the *O. ochengi*-specific assays, OoND5 and OoR1 and OoR5. Assays and *O. ochengi* source DNA are named for the lab where the assay was developed/DNA was isolated: SC = Smith College, TN = Nutman Lab, National Institute of Health, KF = Fischer Lab, Washington University School of Medicine. *** indicates no amplification.

***Onchocerca ochengi* (Oo) DNA**		**Cq values obtained by qPCR Assay**					
**Source**	**Concentration**	**SC OoR01**	**TN OoR01**	**SC OoR05**	**TN OoR05**	**SC OoND5 HEX**	**KF OoND5**
Oo SC	1000 pg	18.89	n/a	24.81	n/a	27.46	27.90
Oo SC	100 pg	25.31	n/a	29.67	n/a	34.57	33.10
Oo SC	10 pg	27.51	n/a	33.75	n/a	36.12	36.10
Oo SC	1 pg	***	n/a	***	n/a	***	
Oo SC	0.1 pg	***	n/a	***	n/a	***	
Oo SC	0.001 pg	***	n/a	***	n/a	***	
Oo TN	1000 pg	22.35	20.97	25.25	23.69	23.95	20.56
Oo TN	100 pg	27.43	24.63	30.30	27.72	29.05	24.82
Oo TN	10 pg	30.72	28.88	35.14	31.70	31.73	28.59
Oo TN	1 pg	35.44	31.51	38.79	35.37	34.47	32.33
Oo TN	0.1 pg	37.00	34.47	***	38.36	37.33	35.15
Oo TN	0.001 pg	***	***	***	***	***	38.96
Oo KF	1000 pg	17.68	17.52	21.71	20.47	22.91	24.29
Oo KF	100 pg	21.31	20.59	25.37	23.71	26.15	28.18
Oo KF	10 pg	25.32	25.11	29.46	28.75	30.42	31.67
Oo KF	1 pg	29.22	28.69	33.75	32.80	34.26	35.65
Oo KF	0.1 pg	33.20	32.35	35.32	35.40	***	***
Oo KF	0.001 pg	36.35	35.26	***	***	***	***
