# Supplementary material for: Optimized strategy for real-time qPCR detection of Onchocerca volvulus DNA in pooled Simulium sp. blackfly vectors
Source: PLoS Negl Trop Dis. 2023 Dec 14;17(12):e0011815. doi: 10.1371/journal.pntd.0011815 (PMC10754622; doi:10.1371/journal.pntd.0011815)
Supplement: S2 Table — (PDF) [file pntd.0011815.s004.pdf]

S2 Table: Calculated mean Cq values for the NIH-O150 Assay and the Ov ND5 Assay, along with OD values for the O150 ELISA Assay performed on 129 blackfly field samples from Cameroon collected for a previous study by Abong *et al.* (2021) [5]. Result of the assay (+ or -) is indicated, based on the cutoff criteria calculated for this study for qPCR, or criteria for ELISA assay, along with the original results obtained using the O150-LAMP and Ov Actin qPCR. Samples are grouped by number of days post MDA treatment: A1-A15 = Day 0, B1-B17 = Day 30, C1-C30 = Day 90, D1-D14 = Day 180, E1-E53 = Day 270. *** indicates no amplification in qPCR assay.

Sample	Mean Cq NIH O150	+/-	Mean Cq Ov ND5	+/-	OD ELISA	+/-	Buea (O-150 LAMP)	Buea (OvActin qPCR)
A1	29.27	+	29.09	+		-	-	-
A2	30.12	+	29.14	+		-	-	-
A3	24.96	+	25.50	+		-	+	+
A4	24.58	+	24.58	+		-	-	-
A5	24.47	+	26.18	+	0.18	+	+	+
A6	31.49	+	31.60	+		-	-	-
A7	30.36	+	31.05	+		-	+	+
A8	25.62	+	24.59	+	0.09	+	+	-
A9	30.68	+	30.73	+		-	-	-
A10	23.85	+	24.64	+	0.41	+	+	+
A11	24.16	+	23.81	+		-	-	-
A12	31.11	+	31.02	+		-	-	-
A13	32.04	+	32.81	+		-	-	-
A14	32.47	+	32.03	+		-	-	-
A15	30.57	+	30.56	+		-	-	-
B1	25.47	+	26.38	+		-	+	+
B2	28.10	+	30.14	+		-	+	-
B3	27.61	+	29.27	+		-	-	-
B4	33.16	-	32.11	+		-	-	-
B5	38.14	-	33.90	-		-	-	-
B6	29.48	+	31.31	+		-	-	-
B7	32.07	+	31.07	+		-	-	-
B8	23.68	+	24.32	+	0.12	+	+	+
B9	31.19	+	30.25	+		-	+	-
B10	25.78	+	26.96	+	0.10	+	+	+
B11	32.98	+	32.29	+		-	-	-
B12	31.34	+	31.39	+		-	-	-
B13	24.56	+	25.82	+	0.14	+	+	+

B14	25.71	+	27.55	+	0.13	+	+	-
B15	25.41	+	26.06	+	0.10	+	-	-
B16	30.46	+	29.38	+		-	-	-
B17	31.96	+	31.37	+	0.13	+	-	-
C1	27.70	+	29.18	+		-	+	+
C2	30.07	+	30.66	+		-	+	+
C3	27.39	+	28.14	+	0.09	+	+	+
C4	30.64	+	31.76	+		-	+	+
C5	28.89	+	28.47	+		-	+	+
C6	35.20	-	35.04	-		-	-	-
C7	30.60	+	31.51	+		-	-	+
C8	30.04	+	31.36	+		-	-	-
C9	31.70	+	31.87	+		-	+	+
C10	28.98	+	29.09	+		-	-	-
C11	33.13	-	31.61	+		-	-	-
C12	32.12	+	31.56	+		-	-	-
C13	27.14	+	28.76	+	0.15	+	-	-
C14	32.26	+	33.03	-		-	-	-
C15	27.65	+	28.90	+		-	-	-
C16	26.17	+	27.73	+	0.10	+	-	-
C17	32.86	+	***	-		-	-	-
C18	28.49	+	31.36	+	0.10	+	-	-
C19	28.48	+	27.94	+		-	-	+
C20	31.39	+	32.63	+		-	-	-
C21	28.42	+	29.67	+		-	-	-
C22	29.03	+	30.69	+	0.13	+	+	+
C23	33.20	-	33.53	-		-	-	-
C24	***	-	35.35	-		-	-	-
C25	29.11	+	32.13	+	0.11	+	+	+
C26	29.51	+	32.14	+	0.10	+	-	-
C27	32.97	+	***	-		-	-	-
C28	33.49	-	32.54	+		-	-	+
C29	37.11	-	36.87	-		-	-	-
C30	33.57	-	36.66	-		-	-	-
D1	***	-	***	-		-	-	-
D2	24.70	+	24.95	+	0.10	+	+	+
D3	***	-	***	-	0.26	+	-	-
D4	22.23	+	22.87	+	0.33	+	+	+

D5	32.86	+	31.52	+	not processed		-	-
D6	39.23	-	***	-		-	-	-
D7	28.18	+	28.55	+		-	-	-
D8	26.69	+	29.12	+	0.17	+	+	+
D9	31.47	+	31.02	+		-	-	-
D10	24.51	+	25.15	+	0.17	+	+	+
D11	27.62	+	28.70	+		-	-	-
D12	32.46	+	33.18	-		-	-	-
D13	25.24	+	27.47	+	0.17	+	-	-
D14	32.65	+	35.27	-		-	-	-
E1	24.31	+	25.53	+	0.11	+	+	+
E2	24.15	+	23.20	+	0.23	+	+	+
E3	25.03	+	24.08	+	0.14	+	-	-
E4	23.79	+	25.05	+	0.27	+	+	+
E5	29.98	+	32.92	+		-	-	-
E6	34.04	-	34.57	-		-	-	-
E7	31.34	+	30.67	+		-	-	-
E8	30.38	+	28.63	+		-	-	-
E9	35.35	-	31.61	+		-	-	-
E10	35.79	-	35.44	-		-	-	-
E11	24.67	+	26.32	+	0.15	+	+	+
E12	35.58	-	35.93	-		-	-	-
E13	26.46	+	28.89	+		-	+	+
E14	35.78	-	34.27	-		-	-	-
E15	31.64	+	33.52	-		-	-	-
E16	***	-	***	-		-	-	-
E17	35.71	-	35.01	-		-	-	-
E18	36.01	-	***	-		-	-	-
E19	35.50	-	35.96	-		-	-	-
E20	25.92	+	27.64	+		-	+	+
E21	24.14	+	25.52	+	0.09	+	+	-
E22	25.56	+	27.77	+	0.10	+	+	-
E23	34.01	-	***	-		-	-	-
E24	36.07	-	***	-		-	-	-
E25	22.86	+	24.30	+	0.19	+	+	+
E26	33.10	-	33.38	-		-	-	-
E27	36.27	-	***	-		-	-	-

E28	23.24	+	25.32	+	0.22	+	+	+
E29	34.31	-	***	-		-	-	-
E30	30.65	+	25.79	+		-	-	-
E31	23.99	+	25.45	+	0.13	+	+	+
E32	32.59	+	34.01	-		-	-	-
E33	23.75	+	24.68	+	0.13	+	+	+
E34	25.77	+	24.64	+	0.14	+	+	+
E35	36.09	-	***	-		-	-	-
E36	27.90	+	29.84	+		-	+	-
E37	31.02	+	32.58	+		-	+	+
E38	33.63	-	***	-		-	-	-
E39	23.55	+	24.36	+	0.25	+	+	+
E40	23.16	+	24.57	+	0.16	+	+	-
E41	22.18	+	24.26	+	0.27	+	+	+
E42	34.52	-	32.62	+		-	-	-
E43	34.61	-	***	-		-	-	-
E44	32.80	+	39.46	-		-	-	-
E45	25.50	+	26.73	+		-	+	+
E46	21.53	+	23.38	+	0.17	+	+	-
E47	28.16	+	30.92	+		-	-	-
E48	23.67	+	25.77	+	0.16	+	+	+
E49	22.38	+	24.21	+	0.22	+	+	+
E50	23.34	+	25.18	+	0.11	+	+	+
E51	30.39	+	30.06	+	not processed		-	+
E52	31.35	+	31.11	+	0.13	+	-	+
E53	35.47	-	32.46	+		-	-	-
