# Supplementary material for: Optimized strategy for real-time qPCR detection of Onchocerca volvulus DNA in pooled Simulium sp. blackfly vectors
Source: PLoS Negl Trop Dis. 2023 Dec 14;17(12):e0011815. doi: 10.1371/journal.pntd.0011815 (PMC10754622; doi:10.1371/journal.pntd.0011815)
Supplement: S3 Table — (PDF) [file pntd.0011815.s005.pdf]

S3 Table. Comparison of OvND5 and NIH O150 qPCR performance in detecting one *O. volvulus* L3 larva spiked into a pool of 100 *Simulium vittatum* blackfly heads at dilutions of 1:20 and 1:50.

Spiking experiment: 1 *O. volvulus* L3 in 100 Blackfly heads – 20 replicates													
1:20 Dilution	OvND5 Assay			NIH O150 Assay			1:50 Dilution	OvND5 Assay			NIH O150 Assay		
Sample	Cq	Mean	SD	Cq	Mean	SD	Sample	Cq	Mean	SD	Cq	Mean	SD
1	28.90	28.52	0.43	30.47	29.96	0.46	1	29.77	29.98	0.19	29.99	29.76	0.21
	28.60			29.86				30.14			29.58		
	28.06			29.56				30.03			29.70		
2	28.41	28.46	0.10	30.07	30.16	0.08	2	30.18	29.96	0.21	30.86	30.31	0.62
	28.58			30.20				29.95			30.42		
	28.40			30.20				29.77			29.64		
3	28.70	28.89	0.18	***	32.63	N/A	3	30.95	30.59	0.37	29.95	30.22	0.27
	28.92			***				30.60			30.48		
	29.06			32.63				30.21			30.24		
4	29.55	29.24	0.87	30.71	31.11	1.07	4	29.58	29.87	1.18	31.39	31.39	0.23
	29.90			30.30				31.17			31.16		
	28.25			32.33				28.87			31.62		
5	28.71	28.98	0.40	29.08	28.75	0.29	5	30.19	30.21	0.17	29.59	29.55	0.04
	28.78			28.53				30.06			29.55		
	29.44			28.64				30.39			29.51		
6	28.79	29.38	0.85	29.87	29.84	0.21	6	31.13	30.62	0.72	30.53	30.36	0.53
	28.99			29.62				29.80			30.78		
	30.36			30.03				30.93			29.76		
7	28.94	28.86	0.62	31.24	31.48	0.51	7	30.25	30.75	0.77	31.18	31.99	0.71
	28.21			32.07				31.64			32.53		
	29.44			31.14				30.35			32.24		
8	29.11	29.38	0.50	***	N/A		8	31.08	31.30	0.87	***	#DIV/0!	#DIV/0!
	29.07			***				32.26			***		
	29.96			***				30.56			***		
9	***	N/A		***	N/A		9	***	35.92	N/A	***	N/A	
	***			***				35.92			***		
	***			***				***			***		
10	30.84	30.02	0.72	30.97	30.89	0.18	10	30.58	31.29	0.63	32.67	32.35	1.35
	29.71			31.01				31.50			30.86		
	29.49			30.68				31.80			33.50		
11	28.82	28.60	0.55	***	N/A		11	29.77	29.98	0.19	29.99	29.76	0.21
	29.00			***				30.14			29.58		
	27.97			***				30.03			29.70		
12	27.79	28.53	0.69	29.63	29.83	0.18	12	30.18	29.96	0.21	30.86	30.31	0.62

	29.17			29.97				29.95			30.42		
	28.63			29.88				29.77			29.64		
13	28.84	29.02	0.18	28.35	28.50	0.18	13	30.95	30.59	0.37	29.95	30.22	0.27
	29.03			28.45				30.60			30.48		
	29.21			28.70				30.21			30.24		
14	28.61	28.64	0.03	***	N/A		14	29.58	29.87	1.18	31.39	31.39	0.23
	28.64			***				31.17			31.16		
	28.66			***				28.87			31.62		
15	28.68	28.46	0.20	30.04	29.61	0.40	15	30.19	30.21	0.17	29.59	29.55	0.04
	28.41			29.26				30.06			29.55		
	28.30			29.54				30.39			29.51		
16	27.42	27.75	0.34	29.14	28.62	0.49	16	31.13	30.62	0.72	30.53	30.36	0.53
	28.11			28.16				29.80			30.78		
	27.73			28.56				30.93			29.76		
17	29.06	29.78	0.81	28.24	28.25	0.32	17	30.25	30.75	0.77	31.18	31.99	0.71
	29.63			28.58				31.64			32.53		
	30.65			27.94				30.35			32.24		
18	28.69	29.39	0.64	31.99	31.95	0.82	18	31.08	31.30	0.87	***	N/A	
	29.54			32.75				32.26			***		
	29.95			31.11				30.56			***		
19	30.12	30.13	0.60	32.36	31.45	1.20	19	***	35.92	#DIV/0!	***	N/A	
	30.73			30.09				35.92			***		
	29.53			31.88				***			***		
20	30.36	29.77	0.54	31.13	30.99	0.77	20	30.58	31.29	0.63	32.67	32.35	1.35
	29.29			30.17				31.50			30.86		
	29.66			31.68				31.80			33.50		
**Assay Mean**	29.04			30.25			**Assay Mean**	30.70			30.74		
**SD**	0.62			1.32			**SD**	1.26			1.12		

Mean and standard deviation for 20 samples run in triplicate are reported for each of the assays. *** indicates no amplification
